# Deficiency of Ninjurin1 attenuates LPS/D‐galactosamine‐induced acute liver failure by reducing TNF‐α‐induced apoptosis in hepatocytes

**DOI:** 10.1111/jcmm.17538

**Published:** 2022-09-07

**Authors:** Min Woo Kim, Ju‐Hee Kang, Hyun Jin Jung, Se Yong Park, Jong‐Ik Hwang, Je Kyung Seong, Yeo Sung Yoon, Seung Hyun Oh

**Affiliations:** ^1^ Department of Anatomy and Cell Biology, College of Veterinary Medicine Seoul National University Seoul South Korea; ^2^ College of Pharmacy Gachon University Incheon South Korea; ^3^ Graduate School of Medicine Korea University Seoul South Korea; ^4^ Korea Mouse Phenotyping Center, College of Veterinary Medicine Seoul National University Seoul South Korea

**Keywords:** cell death, D‐galactosamine, KO mouse, LPS, Ninjurin1, TNFR1

## Abstract

Nerve injury‐induced protein 1 (Ninjurin1, Ninj1) is a membrane protein that mediates cell adhesion. The role of Ninj1 during inflammatory response has been widely investigated in macrophages and endothelial cells. Ninj1 is expressed in various tissues, and the liver also expresses high levels of Ninj1. Although the hepatic upregulation of Ninj1 has been reported in human hepatocellular carcinoma and septic mice, little is known of its function during the pathogenesis of liver diseases. In the present study, the role of Ninj1 in liver inflammation was explored using lipopolysaccharide (LPS)/D‐galactosamine (D‐gal)‐induced acute liver failure (ALF) model. When treated with LPS/D‐gal, conventional Ninj1 knock‐out (KO) mice exhibited a mild inflammatory phenotype as compared with wild‐type (WT) mice. Unexpectedly, myeloid‐specific Ninj1 KO mice showed no attenuation of LPS/D‐gal‐induced liver injury. Whereas, Ninj1 KO primary hepatocytes were relatively insensitive to TNF‐α‐induced caspase activation as compared with WT primary hepatocytes. Also, Ninj1 knock‐down in L929 and AML12 cells and Ninj1 KO in HepG2 cells ameliorated TNF‐α‐mediated apoptosis. Consistent with in vitro results, hepatocyte‐specific ablation of Ninj1 in mice alleviated LPS/D‐gal‐induced ALF. Summarizing, our in vivo and in vitro studies show that lack of Ninj1 in hepatocytes diminishes LPS/D‐gal‐induced ALF by alleviating TNF‐α/TNFR1‐induced cell death.

## 1 INTRODUCTION

Nerve injury‐induced protein 1 (Ninjurin1, Ninj1) is a topically expressed membrane protein and was first reported to be upregulated after nerve damage in Schwann cells.^1^ Ninj1 is composed of 152 amino acids (aa) and contains two extracellular N‐ and C‐terminal domains, two transmembrane domains, and a cytoplasmic domain.^1^, ^2^ In N‐terminal extracellular domain, homophilic adhesion motif (26–37 aa) was identified, and this motif mediates cell to cell adhesion.^3^ The liver is known to express high levels of Ninj1,^3^ and it has been reported that the hepatic expression of Ninj1 is enhanced during sepsis‐induced systemic inflammation.^4^ Furthermore, elevated Ninj1 expression was observed in human hepatocellular carcinoma associated with cirrhosis and viral hepatitis,^5^ implying that Ninj1 might have potential roles in hepatic diseases. However, the roles of Ninj1 during the pathogenesis of liver diseases and the mechanisms responsible are still unknown.

Acute liver failure (ALF), also called fulminant hepatic failure, is a severe clinical syndrome characterized by sudden hepatic dysfunction and high mortality in patients with no history of underlying liver disease.^6^ Massive hepatocyte death that exceeds regenerative capacity constitutes the basis of ALF^7^ and results in hepatic encephalopathy, coagulopathy, jaundice, and multi‐organ failure.^6^ The causes of ALF include viruses, toxins, drugs, shock, and autoimmune hepatitis,^6^, ^8^ and although prognosis is highly dependent on aetiology, liver transplantation is the only therapeutic option in cases of advanced ALF.^9^

The LPS/D‐galactosamine (D‐gal)‐induced acute hepatitis model has been widely used to study ALF.^10^, ^11^ When exposed to LPS/D‐gal, macrophages stimulated by LPS release TNF‐α,^12^ which induces the death of hepatocytes sensitized by D‐gal via tumour necrosis factor receptor 1 (TNFR1).^13^ TNFR1 is ubiquitously expressed in almost all cells, while TNFR2 is expressed at limited levels in immune cells and some other cell types.^14^, ^15^, ^16^ Although the extracellular domains of TNFR1 and TNFR2 have similar structures, only TNFR1 possesses an intracellular death domain, which is required for TNF‐α‐mediated cell death signalling.^17^, ^18^ According to a previous knock‐out (KO) study, TNF‐α KO mice and TNFR1 KO mice were completely resistant to LPS/D‐gal‐induced acute hepatitis, whereas TNFR2‐deficient mice were susceptible,^19^ which demonstrated this model is wholly dependent on the TNF‐α/TNFR1 death signalling pathway.

In this work, we investigated whether Ninj1 plays a crucial role in liver inflammation using an LPS/D‐gal‐induced acute hepatitis murine model. Initially, we observed that loss of Ninj1 attenuated LPS/D‐gal‐induced hepatitis in Ninj1‐deficient mice. Further in vitro and KO mouse experiments with myeloid‐specific or hepatocyte‐specific Ninj1 KO mice were performed to explore the mechanism and specify the cell type involved. These experiments showed that Ninj1 deficiency alleviates LPS/D‐gal‐induced ALF by reducing TNF‐α/TNFR1‐induced apoptosis in hepatocytes.

## 2 MATERIALS AND METHODS

### 2.1 Mice and experimental acute hepatitis

All animal experiments were performed in accordance with protocols approved by the Institutional Animal Care and Use Committee (GIACUC‐R2018020). Mice were maintained under a regular light/dark cycle (12 h light/12 h dark) at 22°C and 60% relative humidity in a specific pathogen‐free facility with free access to food and water. Conventional Ninj1 KO C57BL/6 mice were generated as previously described.^20^, ^21^, ^22^ To establish myeloid‐specific Ninj1 KO mice or hepatocyte‐specific Ninj1 KO mice, Ninj1 floxed mice (KOMP, Ninj1^tm1c[KOMP]Wtsi^) were crossed with Lyz2‐Cre mice (RRID:IMSR_JAX:004781) or Alb‐Cre mice (RRID:IMSR_JAX:018961), respectively.^22^ Pups were weaned at 3 to 4 weeks after birth, and tails were collected for genotyping. Male or female mice (10–12 weeks old) were used for experiments. All mice used in this study were randomly divided into control group or LPS/D‐gal‐treated group after genotyping. The sample size was estimated according to previous publications.^23^, ^24^ The acute hepatitis model was produced by administering LPS (15 μg/kg)/D‐galactosamine (350 mg/kg) in PBS by intraperitoneal injection. After 3.5 h or 5 h, mice were sacrificed and liver tissues and serum were collected for analysis. Serum levels of aspartate aminotransferase (AST) and alanine aminotransferase (ALT) were determined using a HITACHI 7180 analyser. Formalin‐fixed liver tissues were processed to produce paraffin blocks, and sectioned liver tissues were subjected to haematoxylin and eosin (H&E) staining for microscopic observation. Frozen liver tissues were analysed by western blot. Experimental process was conducted without blinding since genotyping was performed before animal experiments.

### 2.2 Genotyping

Mouse genomic DNA (gDNA) was isolated from tails for genotyping. Briefly, tails were dissolved in tail buffer [100 mM Tris, 200 mM NaCl, 5 mM ethylenediaminetetraacetic acid (EDTA), 0.2% sodium dodecyl sulfate (SDS)] containing proteinase K (0.4 mg/ml) overnight at 55°C. After centrifugation to remove debris, supernatants were transferred to isopropanol to precipitate gDNA. Precipitated gDNA pellets were washed once with 70% ethanol, dried, and dissolved in Tris‐EDTA (TE) buffer. gDNA samples were stored at 4°C or −20°C and subsequently subjected to PCR using genotyping primer sets (Table 1). PCR products were detected by agarose gel electrophoresis.

**TABLE 1:** Primers for genotyping

No.	Target	Direction	Primer sequence (5′ to 3′)
1	Conventional Ninj1 KO/WT Ninj1	Forward (WT)	GAG ATA GAG GGA GCA CGA CG
		Forward (Neo)	ACG CGT CAC CTT AAT ATG CG
		Reverse	CGG GTT GTT GAG GTC ATA CTT G
2	Floxed Ninj1/WT Ninj1	Forward	GCT GTA GCT AAA CAA GGT GAC C
		Reverse	CCC AGG GTC TAG GTT CCT G
3	Lyz2‐Cre/WT	oIMR3066	CCC AGA AAT GCC AGA TTA CG
		oIMR3067	CTT GGG CTG CCA GAA TTT CTC
		oIMR3068	TTA CAG TCG GCC AGG CTG AC
4	Alb‐Cre	Forward	GCG GTC TGG CAG TAA AAA CTA TC
		Reverse	GTG AAA CAG CAT TGC TGT CAC TT
5	Floxed Ninj1/Recombined Ninj1	Forward	CTG AGA AGG CGC ATA ACG ATA CC
		Reverse	GGC GAG CTC AGA CCA TAA CTT C

### 2.3 Cells and materials

L929 and HepG2 cell lines were purchased from the Korea Cell Line Bank. AML12 cells were obtained from the American Type Culture Collection (ATCC). L929 and HepG2 cells were cultured in Roswell Park Memorial Institute (RPMI) 1640 medium supplemented with 10% fetal bovine serum (FBS) and penicillin/streptomycin (Welgene, Daegu, Korea). AML12 cells were cultured in Dulbecco's Modified Eagle Medium (DMEM):F12 medium supplemented with 10% FBS, penicillin/streptomycin, insulin, transferrin, selenium, and dexamethasone, according to the manufacturer's instructions. LPS and actinomycin D (ActD) were obtained from Sigma‐Aldrich, and D‐gal was purchased from Cayman. Murine TNF‐α and human TNF‐α were obtained from PeproTech and ProSpec, respectively.

### 2.4 Primary antibodies

Information is included in Appendix S1.

### 2.5 Analysis of mRNA expression data from GEO (Gene Expression Omnibus).

To analyse differential expressions of Ninj1, mRNA expression data sets (GSE17184, GSE38941, and GSE28619) were obtained from the GEO database. The significances of differential Ninj1 expressions between two groups were determined using Welch's *t*‐test.

### 2.6 Immunohistochemistry

Paraffin sections (4 μm) of liver tissues were deparaffinized, rehydrated, and treated with 3% H_2_O_2_ for 30 min at room temperature to block endogenous peroxidase activity. Antigen retrieval was performed in boiling buffer (10 mM citrate, 0.1% Tween 20 and 0.5% EDTA) for 10 min. After 3 washes with PBS‐T (0.05% Tween 20) and a blocking step, sections were incubated with primary antibody overnight at 4°C. Ninjurin1 antibody and cleaved caspase 3 antibody were obtained from AbClon and Cell Signalling Technology(#9661), respectively. The next day, sections were washed with PBS‐T three times, conjugated with secondary antibody for 1 h, and incubated with streptavidin‐horseradish peroxidase solution (Vector Laboratories, PK‐6101). After washing, sections were treated with 3,3′‐diaminobenzidine solution. Evaluations were performed by determining percentages of positively stained cells (None = 0, <1% = 1, 1%–10% = 2, 10%–33% = 3, 33%–66% = 4, 66%–100% = 5).^25^

### 2.7 Enzyme‐Linked Immunosorbent Assay (ELISA)

Information is included in Appendix S1.

### 2.8 RNA interference

Small interfering RNA (siRNA) targeting mouse Ninj1 was obtained from Bioneer (Daejeon, Korea). The sequence of the siRNA was 5′‐GGC AAU GAU UUC GCC UUC U(dTdT)‐3′. To introduce the siRNA into L929 cells, reverse transfection was performed using RNAiMAX (Invitrogen) according to the manufacturer's instructions.

### 2.9 Primary cell isolation

Information is included in Appendix S1.

### 2.10 Generation of the NINJ1 KO HepG2 cell line

Clustered regularly interspaced short palindromic repeats (CRISPR)‐CRISPR‐associated protein 9 (Cas9) control vector and CRISPR‐Cas9 All‐in‐one vector targeting human NINJ1 were purchased from transOMIC Technologies (Huntsville, AL, USA). HepG2 cells were transfected with the CRISPR‐Cas9 control vector or CRISPR‐Cas9 All‐in‐one vector targeting human NINJ1 using Lipofectamine® 2000 transfection reagent, according to the manufacturer's instructions. Transfected HepG2 cells were sorted using turbo red fluorescent protein (tRFP) and SH800Z cell sorter (SONY). Sorted HepG2 cells were then seeded on 96‐well plates for single‐cell colony selection. Western blot analysis was used to confirm NINJ1 KO. We refer to HepG2 cells transfected with CRISPR‐Cas9 control vector expressing NINJ1 as WT and NINJ1‐deficient cells as NINJ1 KO.

### 2.11 Establishment of the Ninj1 KD AML12 cell line

Lentiviral vectors containing control short hairpin RNA construct (shCon) or shRNA construct against mouse Ninj1 (shNinj1) were obtained from Open Biosystems. AML12 cells were treated with lentiviral vectors to establish AML12‐shCon or AML12‐shNinj1 cells, according to the manufacturer's instructions.

### 2.12 Cell culture and treatments

All cell lines and primary cells were cultured at 37°C in a 5% CO_2_ humidified chamber. Primary hepatocytes isolated from WT or Ninj1 KO mice were treated with murine TNF‐α (20 ng/ml) and ActD (500 ng/ml) for 6 h. L929 cells were transfected with scrambled siRNA or siRNA targeting Ninj1, as described above, and then, exposed to murine TNF‐α (20 ng/ml) and ActD (250 ng/ml) for 6 h. AML12 cells were treated with murine TNF‐α (20 ng/ml) and ActD (500 ng/ml) for 8 to 10 h. WT and Ninj1 KO HepG2 cells were incubated with human TNF‐α (25 ng/ml) and ActD (500 ng/ml) for 8 to 10 h. After treatments, cells were analysed by western blot.

### 2.13 Western blot analysis

Western blot was performed as described in a previous report.^26^ Detailed information is included in Appendix S1.

### 2.14 MTT assay

Information is included in Appendix S1.

### 2.15 Immunocytochemistry

L929 and HepG2 cells were seeded on coverslips. After removing culture media and washing with PBS, cells were fixed with 4% paraformaldehyde for 5 min, permeabilized with 0.2% Tween 20 for 15 min, and blocked with antibody diluent solution. After overnight incubation with primary antibodies (Ninj1, AbClon, 1:100, TNFR1, sc‐8436, 1:100) at 4°C, cells were treated for 4 h with secondary antibodies conjugated with fluorescent dye (FITC or Texas Red) against each primary antibody. Finally, stained cells were mounted with DAPI mounting solution and observed under a confocal microscope.

### 2.16 Co‐immunoprecipitation

WT or NINJ1 KO HepG2 cells were lysed with buffer (150 mM NaCl, 20 mM Tris–HCl, pH 8.0, 10% glycerol, 0.3% Nonidet P‐40) containing phosphatase inhibitor and protease inhibitor. After 1 h incubation on ice, cell lysates were obtained by centrifugation for 10 min at 10,000 *g*. Lysates were incubated with antibody against TNFR1 (sc‐8436) at 4°C overnight. Then, protein A/G PLUS‐agarose beads (sc‐2003) were applied according to the manufacturer's instructions. Immunoblotting was performed as described above.

### 2.17 Statistical analysis

Results are presented as means ± standard errors of means (SEMs). The analysis was conducted using the with GraphPad Prism 6 by applying unpaired student *t*‐test. Statistical significance was accepted for *p* values <0.05.

## 3 RESULTS

### 3.1 Expressions of Ninj1 in the murine liver inflammation model and hepatitis patients

**FIGURE 1:**
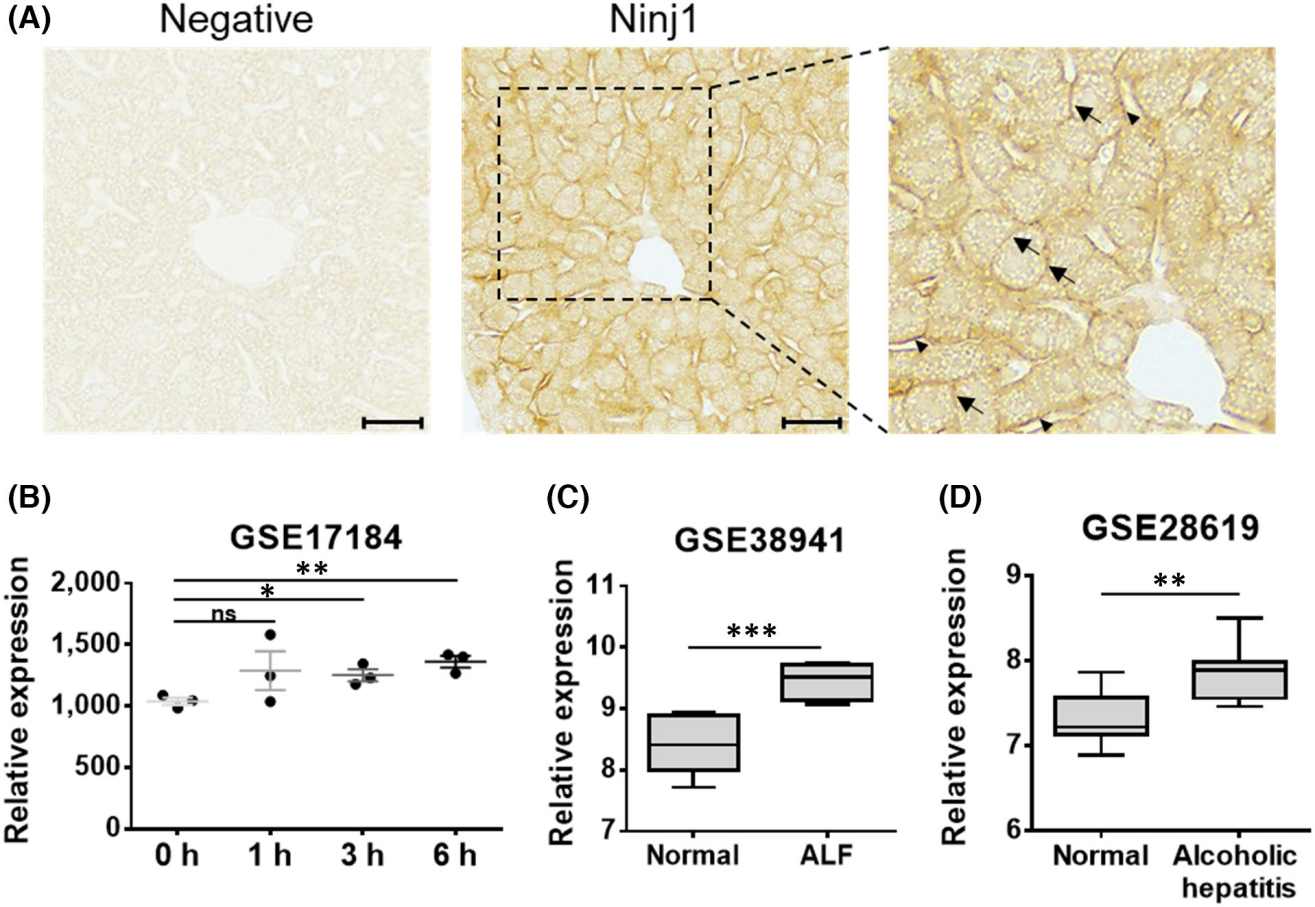
Ninj1 expression in hepatitis and human patients. (A) IHC was conducted to detect Ninj1 in normal murine liver tissues (left‐negative control, right‐Ninj1 IHC, boxed area‐enlarged image, scale bar 50 μm, arrow: surface of a hepatocyte, arrowhead: surface of sinusoid). (B) Analysis of Ninj1 mRNA expression in concanavalin A‐induced murine hepatitis model (GSE17184). (C, D) Analysis of Ninj1 mRNA expressions in ALF (C, GSE38941) and alcoholic hepatitis patients (D, GSE28619). (B–D) Gene expression data (GSE17184, GSE38941, and GSE28619) were obtained from the GEO database. Welch's *t*‐test was used to determine the significances of differences (ns, not significant; *p < 0.05; **p < 0.01; ***p < 0.001).

Before investigating whether Ninj1 is associated with the development of hepatitis, we confirmed the expression pattern of Ninj1 in normal liver tissue. Immunohistochemistry (IHC) showed Ninj1 was expressed in normal mouse liver, as previously reported,^3^ and that surfaces of hepatocytes and sinusoid exhibited positive signals (Figure 1A). We then investigated Ninj1 expressions using GEO public data sets obtained from mice with experimental hepatitis and human ALF or alcoholic hepatitis patients. In the GSE17184 data set, Ninj1 gene expression level was higher in liver tissues from concanavalin A‐treated mice (3 and 6 h) than in non‐treated controls (Figure 1B). As regards human data, hepatitis B virus‐associated ALF (GSE38941) and alcoholic hepatitis (GSE28619) patients exhibited significant increases in Ninj1 expression in liver tissues as compared with normal controls (Figure 1C,D). These results suggest the possibility that Ninj1 have a role on development of liver inflammation and ALF.

### 3.2 Deletion of Ninj1 in mice attenuates LPS/D‐gal‐induced hepatitis

**FIGURE 2:**
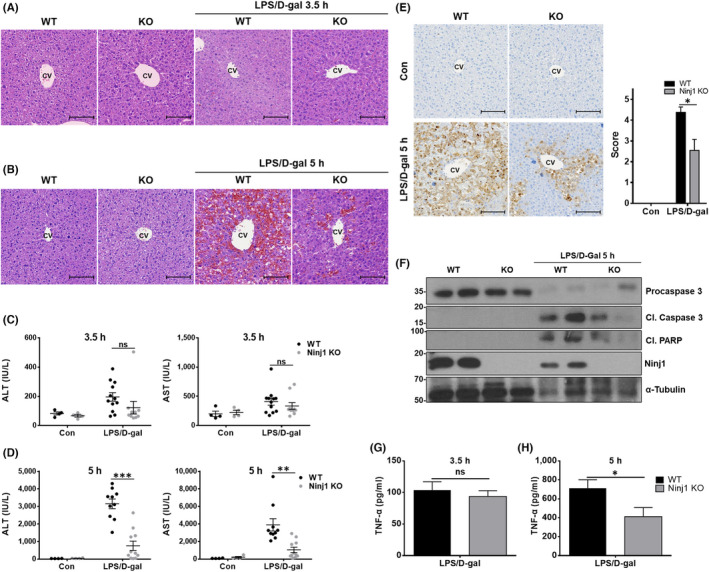
Deletion of Ninj1 in mice attenuates LPS/D‐gal‐induced ALF. (A–G) LPS (15 μg/kg)/D‐gal (350 mg/kg) was intraperitoneally injected into WT and Ninj1 KO mice (n = 4–12). After 3.5 h (A) or 5 h (B), mice were sacrificed and liver tissues were subjected to H&E staining for microscopic observations (scale bar 100 μm, CV: central vein). (C, D) ALT and AST levels were measured in the serum samples of mice exposed to LPS/D‐gal for 3.5 h (C) or 5 h (D). (E) Liver tissues of WT and Ninj1 KO mice treated or not with LPS (15 μg/kg)/D‐gal (350 mg/kg) for 5 h were subjected to IHC using cleaved caspase 3 antibody (Scale bar 100 μm, CV: central vein). (F) Western blotting was conducted on liver tissues from WT and Ninj1 KO mice to examine the cleavage of caspase 3 and PARP. (G, H) ELISA was used to measure TNF‐α levels in serum samples obtained from WT and Ninj1 KO mice exposed to LPS/D‐gal for 3.5 h (G) or 5 h (H). Statistical analysis was performed using the unpaired *t*‐test (ns, not significant; *p < 0.05, **p < 0.01; ***p < 0.001).

To determine whether Ninj1 plays a crucial role in liver inflammation, WT and conventional Ninj1 KO male mice were treated with LPS (15 μg/kg)/D‐gal (350 mg/kg) intraperitoneally, and then sacrificed 3.5 h or 5 h later. When observed microscopically, LPS/D‐gal administration for 3.5 h induced almost no change in the liver tissues of WT and Ninj1 KO mice, although several WT and Ninj1 KO mice showed immune cell infiltration (Figure 2A). On the other hand, exposure to LPS/D‐gal for 5 h induced severe hepatic inflammation in WT mice (Figure 2B), but interestingly, Ninj1 KO mice showed milder hepatitis than WT mice. To evaluate the severity of hepatitis, we measured serum ALT and AST levels, which are widely used clinically and are known as good markers reflecting death of hepatocytes.^27^ LPS/D‐gal treatment for 3.5 h elevated serum ALT and AST in both WT and Ninj1 KO mice (Figure 2C). Hepatitis‐induced Ninj1 KO mice tended to have lower ALT and AST levels than WT mice, but this was not significant. However, after treatment for 5 h, ALT and AST levels increased considerably in WT mice to levels significantly higher than in Ninj1 KO mice (Figure 2D). IHC was also conducted on liver tissues to evaluate apoptotic cell death, which is a pathologic feature of the LPS/D‐gal‐induced ALF model.^28^, ^29^ In agreement with H&E staining and serum biochemistry results, the number of cleaved caspase 3‐positive cells was significantly lower in the liver tissues of Ninj1 KO mice than in those of WT mice (Figure 2E), and this result was confirmed by western blotting (Figure 2F). In addition, ELISA was performed on serum samples to detect TNF‐α, which is the main cytokine responsible for the apoptosis of hepatocytes in LPS/D‐gal‐treated mice.^19^ In the 3.5 h treatment set, no difference was observed between the serum TNF‐α levels of WT and Ninj1 KO mice, while a significant difference was observed in the 5 h treatment set (Figure 2G,H). Collectively, these observations show that Ninj1 deficiency in mice ameliorates the severity of LPS/D‐gal‐induced ALF. This finding also was confirmed in WT and Ninj1 KO female mice (Figure S1A,B). Consistently, deletion of Ninj1 in female mice reduced the liver inflammation and serum ALT and AST levels observed in WT female mice after LPS/D‐gal treatment for 5 h.

### 3.3 Myeloid‐specific ablation of Ninj1 does not reduce the severity of LPS/D‐gal‐induced liver injury

**FIGURE 3:**
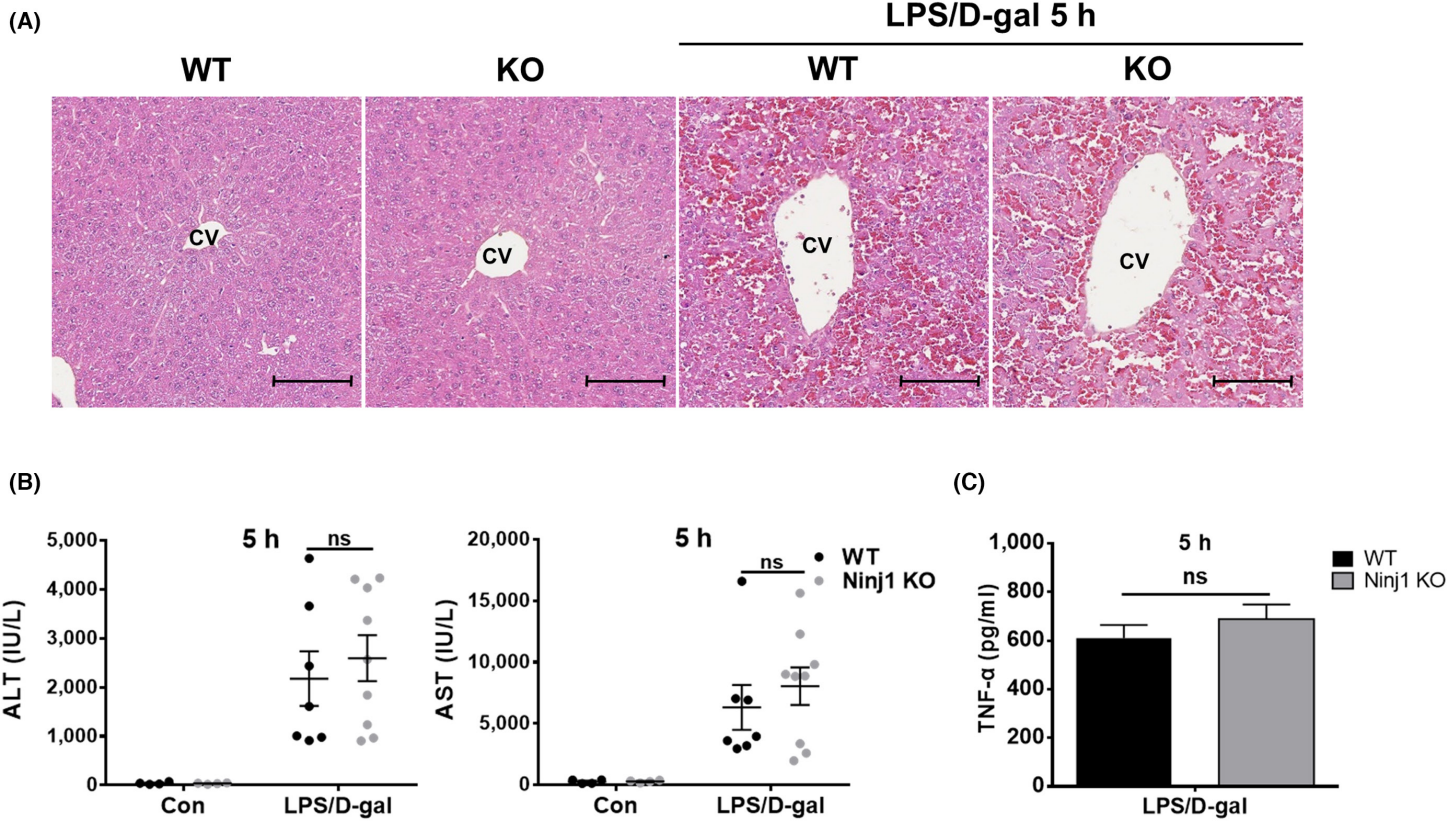
Myeloid‐specific Ninj1 knock‐out mice does not attenuate LPS/D‐gal‐induced acute liver inflammation. (A–C) WT and myeloid‐specific Ninj1 KO mice were administered LPS (15 μg/kg)/D‐gal (350 mg/kg) intraperitoneally (*n* = 4–9), and 5 h later mice were sacrificed and liver tissues were subjected to histological examination (A, Scale bar 100 μm, CV: central vein). (B) ALT and AST levels were measured in serum samples. (C) ELISA was used to evaluate TNF‐α serum levels. Statistical analysis was performed using the unpaired *t*‐test (ns, not significant).

The pathologic mechanism of LPS/D‐gal‐induced ALF primarily involves the activation of macrophages by LPS. Subsequently, these macrophages release TNF‐α, which induces the death of hepatocytes sensitized by D‐gal. Therefore, we first examined whether Ninj1 expressed by macrophages participates in the pathogenesis of LPS/D‐gal‐induced ALF. Conditional Ninj1 KO mice carrying Ninj1‐deficient myeloid cells were established as described previously.^22^ Ninj1 floxed mice with loxP targeted exon 2 of Ninj1 were crossed with Lyz2‐Cre mice to generate myeloid‐specific Ninj1 KO mice. WT mice (Ninj1^+/+^; Lyz2‐Cre/+) and myeloid‐specific Ninj1 KO mice (Ninj1^floxed/floxed^; Lyz2‐Cre/+) were subjected to LPS/D‐gal‐induced hepatitis. Administration of LPS/D‐gal for 5 h caused hepatitis in WT and myeloid‐specific Ninj1 KO mice, and no difference in severity was observed by histological analysis (Figure 3A). Also, no differences in serum ALT, AST (Figure 3B), or TNF‐α (Figure 3C) levels were observed between LPS/D‐gal‐exposed WT and myeloid‐specific Ninj1 KO mice. Taken together, these results suggest macrophage Ninj1 does not play a crucial role in the pathogenesis of LPS/D‐gal‐induced hepatitis and that TNF‐α release by macrophages is not a primary factor of the phenotype of conventional Ninj1 KO mice.

### 3.4 Loss of Ninj1 decreases TNF‐α‐induced hepatocyte death

**FIGURE 4:**
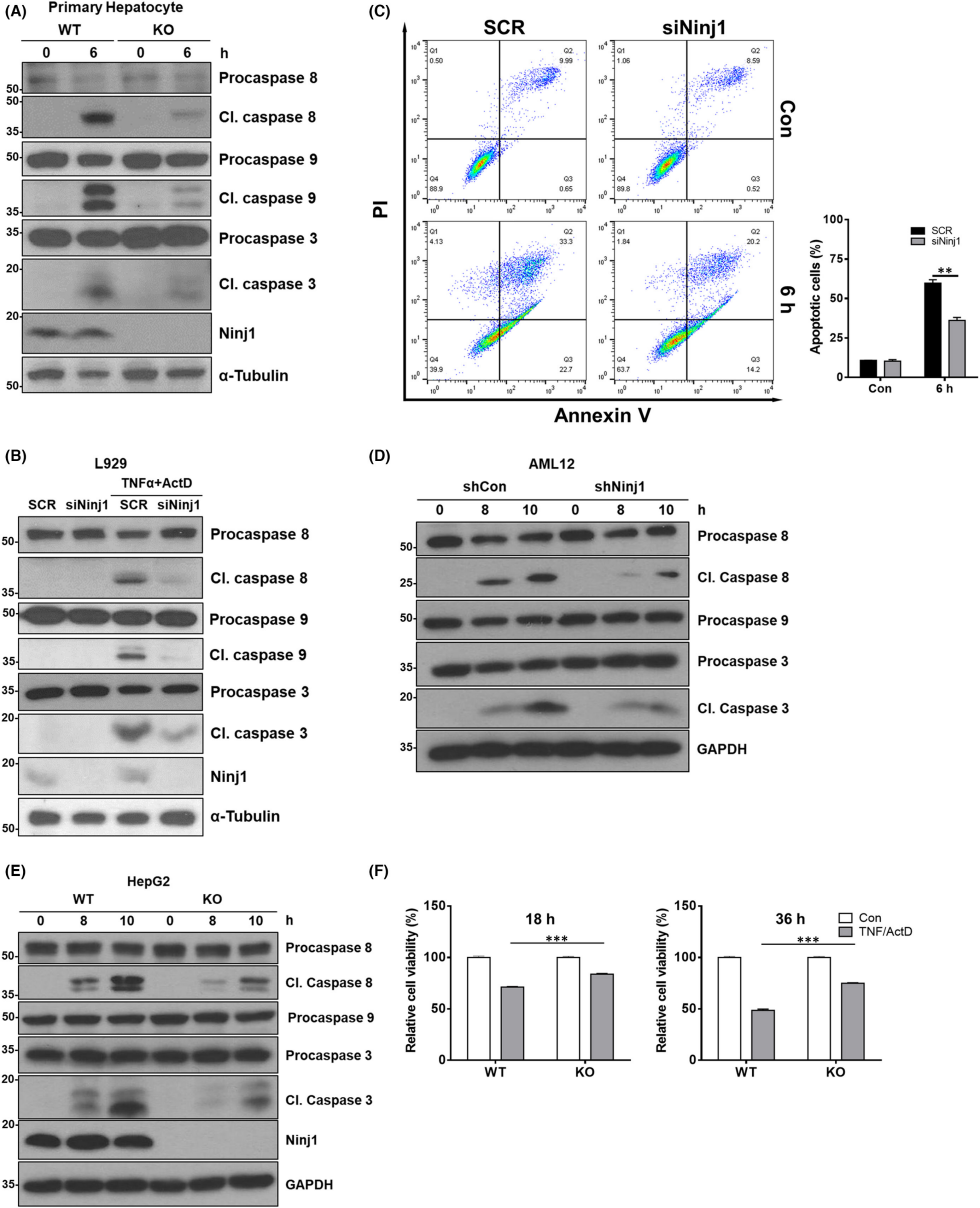
Loss of Ninj1 decreases TNF‐α‐induced cell death in hepatocytes. (A, B, D, and E) Western blot analysis was performed to evaluate caspase cascade activation by TNF‐α. (A) Primary hepatocytes were isolated from WT or Ninj1 KO mice and then exposed to TNF‐α (20 ng/ml)/ActD (500 ng/ml) for 6 h. (B) L929 cells transfected with scrambled siRNA (SCR) or siRNA targeting Ninj1 (siNinj1) were treated with TNF‐α (20 ng/ml)/ActD (250 ng/ml) for 6 h. (C) Apoptosis of L929 cells was assessed by PI/Annexin V staining (*n* = 3). (D) AML12‐shCon and AML12‐shNinj1 cells were treated with TNF‐α (20 ng/ml)/ActD (500 ng/ml) for the indicated times. (E) TNF‐α (25 ng/ml)/ActD (500 ng/ml) was administered to WT and Ninj1 KO HepG2 cells for the indicated times. (F) Viabilities of cells treated with TNF‐α/ActD were assessed using an MTT assay. WT or Ninj1 KO HepG2 cells were exposed to TNF‐α/ActD for 18 or 36 h (*n* = 6). Statistical analysis was performed using the unpaired *t*‐test (***p < 0.001).

Since Ninj1 KO in myeloid cells did not attenuate hepatitis caused by LPS/D‐gal, we investigated whether loss of Ninj1 influences TNF‐α‐induced hepatocyte death. Primary hepatocytes were isolated from WT and conventional Ninj1 KO mice treated with TNF‐α (20 ng/ml) and ActD (500 ng/ml) for 6 h, which sensitizes cells to TNF‐α by inhibiting RNA synthesis.^30^ Western blot revealed weaker caspase activations (caspase 8, 9, and 3) in Ninj1 KO primary hepatocytes than in WT primary hepatocytes (Figure 4A). To confirm this, TNF‐α‐sensitive L929 cells, murine fibroblasts, were transfected with scrambled siRNA or siRNA targeting Ninj1 and then cotreated with murine TNF‐α (20 ng/mL) and ActD (250 ng/mL) for 6 h. In line with primary hepatocyte observations, transient KD of Ninj1 using siRNA decreased caspase activation and apoptosis (Figure 4B,C). In addition, Ninj1 KD AML12 cells, mouse hepatocytes, were generated using lentiviral vector and co‐exposed to TNF‐α (20 ng/ml) and ActD (500 ng/ml) for prescribed times. We observed Ninj1 KD delayed and reduced the cleavage of caspase 8 and 3 in AML12 cells (Figure 4D). Finally, these results were checked in human cell line using CRSPR Cas9‐derived NINJ1 KO HepG2 cells. When WT or NINJ1 KO HepG2 cells were co‐incubated with human TNF‐α (25 ng/ml) and ActD (500 ng/ml) for the indicated times to induce caspase activation, the cleavage of caspase 8 and 3 was reduced by Ninj1 KO (Figure 4E). Also, TNF‐α/ActD‐treated NINJ1 KO HepG2 cells exhibited greater viability than WT HepG2 cells (Figure 4F). Collectively, these observations of primary cells or multiple cell lines strongly suggest that Ninj1 down‐regulation or deficiency blunts the TNF‐α/TNFR1‐mediated apoptosis signal.

### 3.5 Ninj1 KD and KO do not influence TNFR1 complex I signalling or the expressions of TNFR1 or anti‐apoptotic genes

**FIGURE 5:**
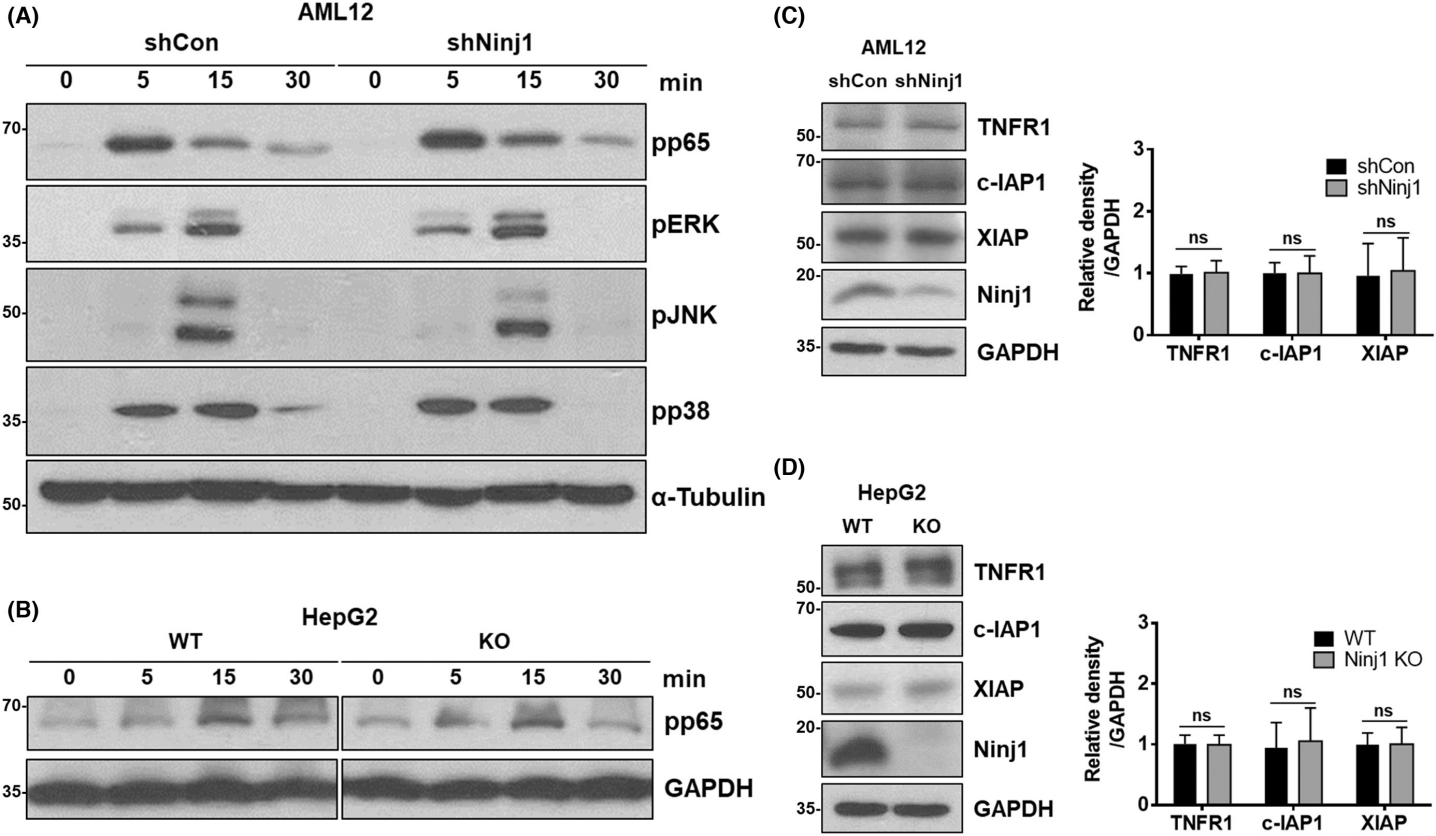
Loss of Ninj1 does not influence TNFR1 complex I signalling or the expressions of TNFR1 or anti‐apoptotic genes. (A, B) TNFR1 complex I signalling was investigated by western blotting. (A) AML12‐shCon and AML12‐shNinj1 cells were treated with murine TNF‐α (20 ng/ml) for the indicated times. (B) WT and Ninj1 KO HepG2 cells were treated with human TNF‐α (25 ng/ml) for the indicated times. (C, D) The protein expressions of TNFR1 and those of anti‐apoptotic genes in AML12 cells (shCon and shNinj1) and HepG2 cells (WT and Ninj1 KO) were examined by western blotting. Statistical analysis was performed using the unpaired *t*‐test (*n* = 3, ns, not significant).

**FIGURE 6:**
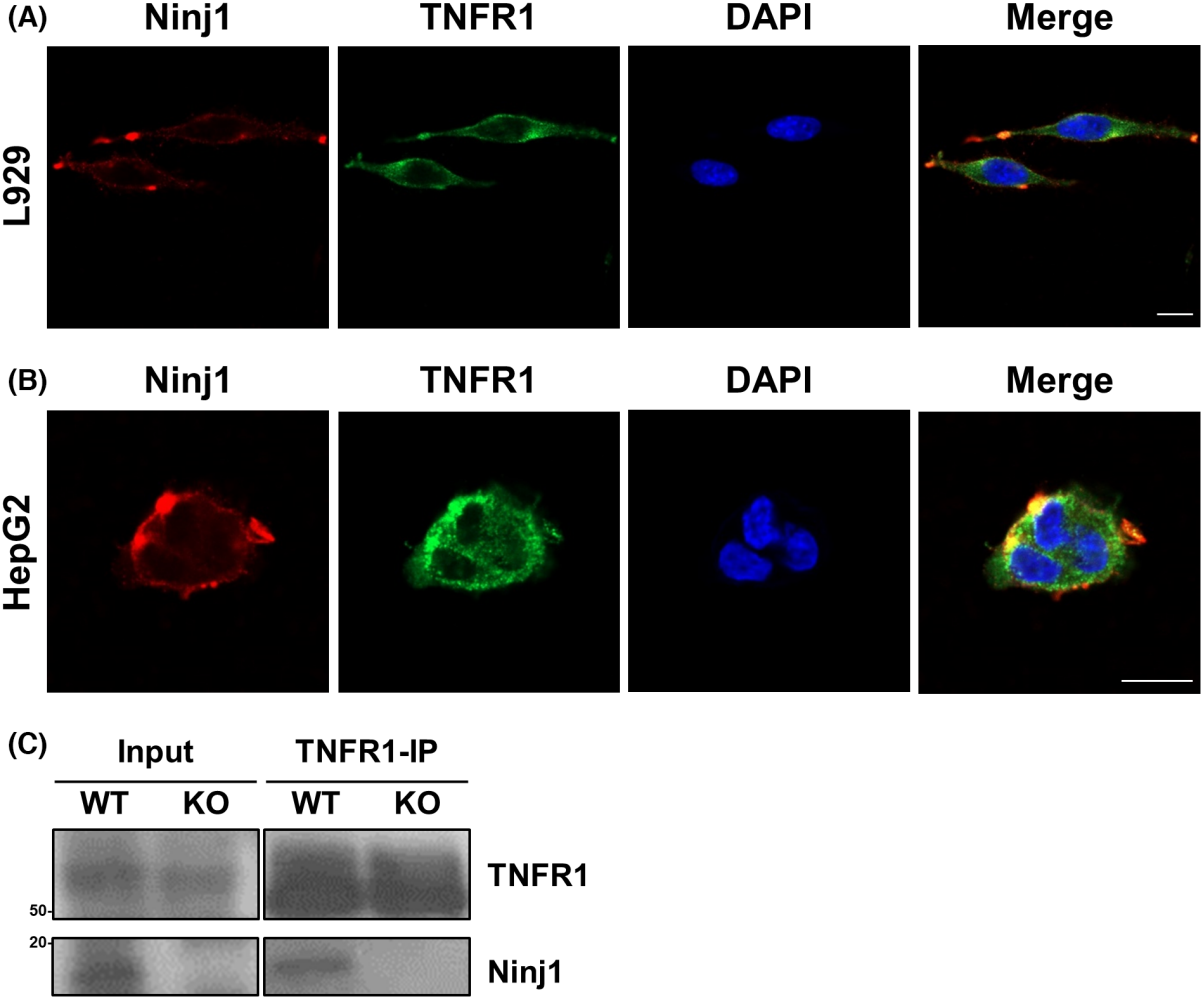
Co‐localization of TNFR1 and Ninj1. (A, B) Co‐localization of TNFR1 (green) and Ninj1 (red) in L929 (A) and HepG2 (B) cells was investigated by immunocytochemistry (Scale bar 10 μm). Nuclei were stained with DAPI (blue). (C) Co‐immunoprecipitation was conducted using anti‐TNFR1 antibody in WT and Ninj1 KO HepG2 cells, and immunoblotting was performed using anti‐TNFR1 and anti‐Ninj1 antibodies.

In vitro KD and KO experiments revealed that loss of Ninj1 dampened the TNFR1‐mediated apoptosis signalling pathway induced by complex II formation. Therefore, we examined whether Ninj1 regulates TNFR1 complex I signalling. AML12‐shCon or AML12‐shNinj1 cells were treated with TNF‐α for prescribed times. Western blotting showed no difference between these two cell types in terms of the phosphorylations of p65, ERK, JNK, and p38 (Figure 5A). Also, WT and NINJ1 KO HepG2 cells were exposed to TNF‐α for prescribed times, and NINJ1 KO was found not to regulate TNFR1 complex I signalling (Figure 5B). These results indicate that loss of Ninj1 did not affect the TNFR1 complex I signalling pathway. To address the possibility that Ninj1 might regulate the expression of TNFR1 or anti‐apoptotic genes, western blot was conducted on AML12 and HepG2 cells. The results obtained showed the expressions of TNFR1 and anti‐apoptotic genes (c‐IAP1 and XIAP) were not altered by Ninj1 KD or KO in either cell type (Figure 5C,D). These results suggest that the role of Ninj1 in TNF‐α‐induced caspase activation is irrelevant to the expression levels of TNFR1 or anti‐apoptotic genes. To investigate the possibility of direct or indirect interaction between TNFR1 and Ninj1, we performed a co‐localization study in L929 and HepG2 cells (Figure 6A,B). Partial co‐localization spots were detected in both cell lines. Furthermore, Ninj1 was detected by co‐immunoprecipitation analysis using TNFR1 antibody in WT HepG2 cells (Figure 6C), which indicating possibility that TNFR1 and Ninj1 interact.

### 3.6 Hepatocyte‐specific Ninj1 KO decreases the severity of acute liver inflammation induced by LPS/D‐gal

**FIGURE 7:**
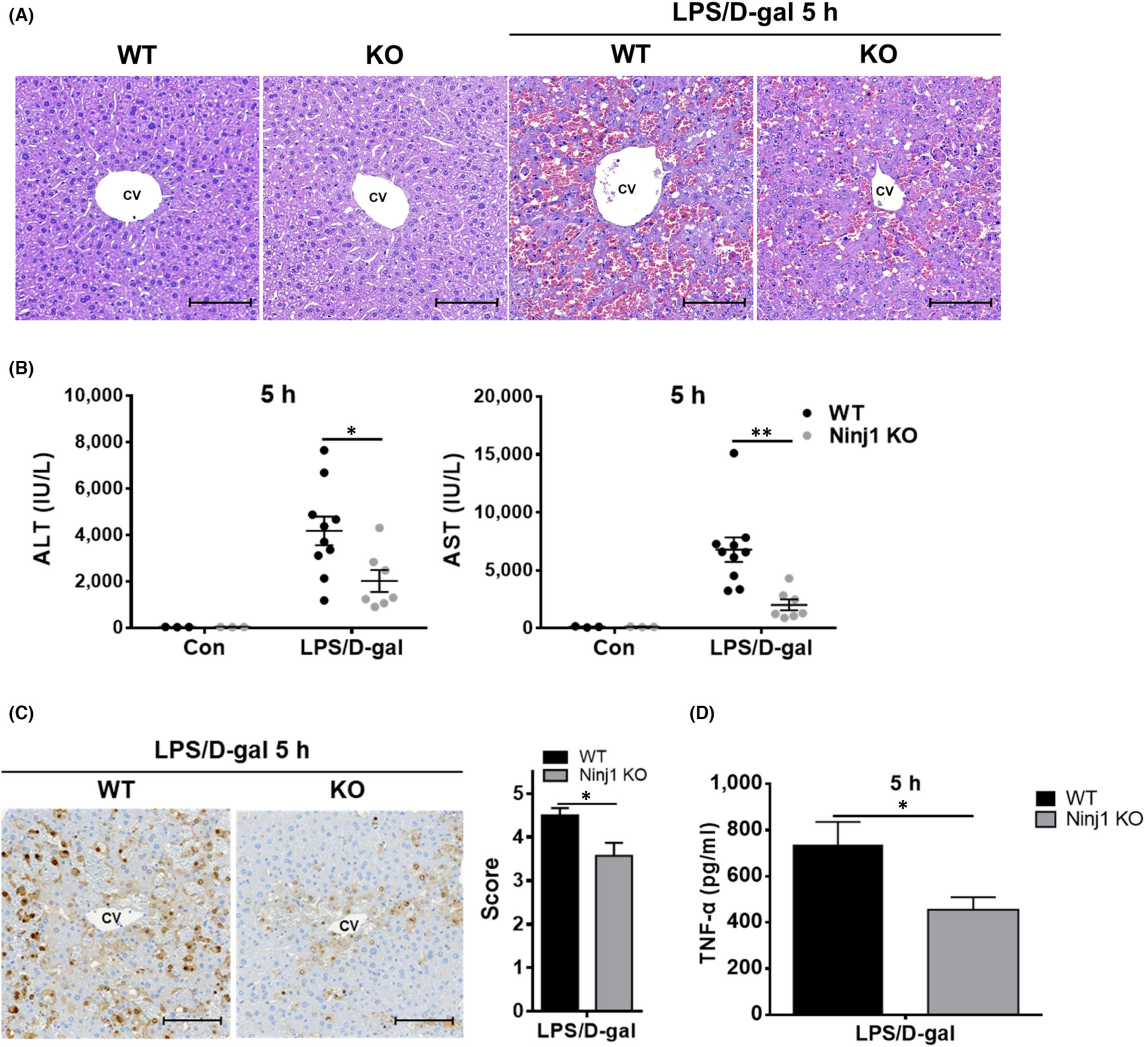
Hepatocyte‐specific Ninj1 ablation suppresses LPS/D‐gal‐induced hepatitis and caspase activation in mice. (A–D) Acute hepatitis was induced by injecting WT and hepatocyte‐specific Ninj1 KO mice (*n* = 3–10) with LPS (15 μg/kg)/D‐gal (350 mg/kg) intraperitoneally. Mice were sacrificed 5 h later, and liver tissues were stained with H&E A (Scale bar 100 μm, CV: central vein). (B) Serum ALT and AST levels were measured in WT and hepatocyte‐specific Ninj1 KO mice. (C) Caspase 3 activation in liver tissues was evaluated by IHC. (D) Serum TNF‐α levels in WT and hepatocyte‐specific Ninj1 KO mice treated with LPS/D‐gal. Statistical analysis was performed using the unpaired *t*‐test (*p < 0.05).

Considering the pathogenesis of LPS/D‐gal‐induced liver inflammation and our in vitro experiment data, we hypothesized that Ninj1 in hepatocytes plays a crucial role in the pathogenesis of LPS/D‐gal‐induced ALF. To investigate this hypothesis, hepatocyte‐specific Ninj1 KO mice were generated by crossing Ninj1 floxed mice (Ninj1^floxed/floxed^) and Alb‐Cre mice (Figure S2A,B). Liver‐specific allele recombination was confirmed by genotyping gDNA from tail, heart, lung, kidney, spleen, and liver tissues (Figure S2C) and by determining Ninj1 protein levels in kidney, spleen, and liver tissues (Figure S2D). To study the function of Ninj1 in hepatocytes in a background of LPS/D‐gal‐induced ALF, WT (Ninj1^+/+^; Alb‐Cre/+) and hepatocyte‐specific Ninj1 KO mice (Ninj1^floxed/floxed^; Alb‐Cre/+) were exposed to LPS/D‐gal for 5 h. Interestingly, LPS/D‐gal‐induced inflammation was less severe in hepatocyte‐specific Ninj1 KO mice than in WT mice (Figure 7A), and this observation was supported by significantly different ALT and AST levels in WT and hepatocyte‐specific KO mice treated with LPS/D‐gal (Figure 7B). To compare caspase activation in the liver tissues of WT and hepatocyte‐specific Ninj1 KO mice, IHC was conducted for cleaved caspase 3. We observed that hepatocyte‐specific Ninj1 ablation decreased caspase 3 cleavage in LPS/D‐gal‐exposed livers (Figure 7C). Furthermore, serum TNF‐α levels were lower in hepatocyte‐specific Ninj1 KO mice than in WT mice (Figure 7D). These results obtained from the hepatocyte‐specific conditional KO study demonstrated that in hepatocytes Ninj1 plays a crucial role in the pathogenesis of LPS/D‐gal‐induced ALF.

## 4 DISCUSSION

Ninj1 is a small membrane protein involved in cell adhesion, and its expression is induced by nerve injury and inflammatory response.^1^ Furthermore, Ninj1 was reported to be extensively expressed in various adult and embryo tissues,^3^ and subsequent studies demonstrated its involvement in biological processes as diverse as migration, inflammation, metastasis, development, and growth.^1^, ^4^, ^20^, ^31^, ^32^, ^33^ The liver is one of the organs that express Ninj1, and the upregulation of Ninj1 in liver has been reported in human hepatocellular carcinoma and septic mice,^4^, ^5^ which suggests Ninj1 may play roles in the pathogenesis of hepatic diseases. Nevertheless, little is known about the function of Ninj1 during the development of liver diseases.

The role of Ninj1 in inflammatory diseases has been highlighted by recent studies,^4^, ^20^, ^22^, ^31^, ^34^ and our analysis of public GEO data sets revealed Ninj1 expression is upregulated in mice subjected to experimental hepatitis and in human ALF or alcoholic hepatitis patients, which indicates Ninj1 is involved in liver inflammation. Thus, we studied the function of Ninj1 in liver using an LPS/D‐gal‐induced ALF model of inflammation.

In this study, we report for the first time that loss of Ninj1 in mice markedly mitigates LPS/D‐gal‐induced ALF. In an initial animal experiment using conventional Ninj1 KO mice, histology revealed only mild inflammation in the liver tissues of LPS/D‐gal‐exposed Ninj1 KO mice as compared with that observed in LPS/D‐gal‐exposed WT mice. Likewise, levels of ALT, AST, TNF‐α, and caspase 3 activation were significantly lower in LPS/D‐gal‐treated Ninj1 KO mice, and this phenotype was also observed in their female counterparts. Furthermore, the results of our conventional KO study concurred with those of a previous study on the role of Ninj1 in DSS‐induced colitis, which demonstrated that Ninj1 in macrophages contributes to colitis by increasing pro‐inflammatory cytokine expressions.^22^ Also, TNF‐α secreted by macrophage is a main player in LPS/D‐gal‐induced hepatitis model.^19^ Based on our observation that serum TNF‐α levels of Ninj1 conventional KO mice were lower than those of WT mice after 5 h of LPS/D‐gal treatment, we supposed lack of Ninj1 in myeloid‐lineage cells, including macrophages, had attenuated the severity of acute hepatitis by reducing TNF‐α production. Unexpectedly, we found serum ALT, AST, and TNF‐α levels were similar in mice harbouring myeloid cells deficient in Ninj1 and WT mice in LPS/D‐Gal‐induced ALF model. Although the pro‐inflammatory role of Ninj1 in macrophages has been reported on several occasions,^4^, ^22^, ^34^ loss of Ninj1 in myeloid cells did not attenuate LPS/D‐gal‐induced ALF. A possible explanation can be extracted from the individual characteristics of disease models. The development of LPS/D‐gal‐induced ALF occurs more rapidly than in other inflammatory disease models, requiring only several hours. In addition, LPS‐stimulated macrophages generate soluble TNF‐α by releasing pre‐existing membrane TNF‐α via TNF‐α converting enzyme (TACE).^35^, ^36^ Thus, previously reported functions of Ninj1 on inflammatory signalling pathways and pro‐inflammatory gene expressions in macrophages appear uninfluential in the LPS/D‐gal‐induced ALF model.

During the pathogenesis of LPS/D‐gal‐induced acute liver inflammation, TNF‐α released by macrophages induces D‐gal‐sensitized hepatocyte death via TNFR1.^13^, ^19^ Thus, we explored the TNF‐α/TNFR1 death signalling pathway by conducting in vitro experiments. Remarkably, TNF‐α/ActD treatment reduced caspase 8, 9, and 3 activations in primary hepatocytes isolated from Ninj1 KO mice more than in primary hepatocytes from WT mice. Furthermore, transient KD of Ninj1 in L929 murine fibroblast cells and stable KD of Ninj1 in AML12 murine hepatocytes consistently suppressed TNF‐α/ActD‐induced caspase activation, and a similar consistent phenomenon was observed in NINJ1 KO HepG2 human cells. These findings strongly support the involvement of Ninj1 in the TNF‐α/TNFR1 death signal.

In all of the cell types used in the present study, that is, primary hepatocytes and L929, AML12, and HepG2 cells, KD or KO of Ninj1 reduced the TNF‐α‐induced cleavage of caspase 8, which is activated immediately after complex II formation.^37^, ^38^ This suggest Ninj1 influences complex II formation after TNF‐α binds to TNFR1. On the other hand, TNFR1 complex I signalling was not influenced by Ninj1 KD or KO in AML12 or HepG2 cells. Also, the expressions of TNFR1 and anti‐apoptotic genes were unaffected by Ninj1 KD or KO. Collectively, Ninj1 only regulates the death signal mediated by TNFR1 complex II formation without affecting the expressions of TNFR1 or anti‐apoptotic genes, which suggests Ninj1 regulates the intracellular complex II signalling pathway by directly or indirectly interacting with TNFR1. Although the mechanism how Ninj1 specifically regulates TNFR1 complex II signalling was not clarified, our co‐localization and co‐immunoprecipitation analysis results provide the possibility of molecular interaction between TNFR1 and Ninj1.

In agreement with our in vitro data, hepatocyte‐specific Ninj1 KO mice showed less severe LPS/D‐gal‐induced ALF than WT mice, as evidence by histological observations and serum chemistry. Furthermore, after LPS/D‐gal exposure cleaved caspase 3 positive cells were significantly fewer in the liver tissues of hepatocyte‐specific KO mice than in those of WT mice. These outcomes support our in vitro results and show that Ninj1 expressed in hepatocytes participate in the pathogenesis of LPS/D‐gal‐induced ALF.

Having considered the experimental results obtained from conventional Ninj1 KO, myeloid‐specific Ninj1 KO, and hepatocyte‐specific Ninj1 KO mice, we suggest that hepatocyte Ninj1 is required for the development of LPS/D‐gal‐induced ALF and that macrophage Ninj1 does not play a critical role in the pathogenesis of ALF. We believe our in vivo findings improve understanding of the pathogenesis of ALF and provide the possibility that pharmacological inhibition of Ninj1 in hepatocytes might provide a therapeutic strategy.

In this study, genetic ablation of Ninj1 was utilized to study the functions of Ninj1 in an LPS/D‐gal‐induced ALF model, whereas in another studies, a Ninj1 blocking peptide corresponding to the adhesion motif of Ninj1 was used.^4^, ^29^ In a previous study, the authors treated endothelial cells and macrophages with Ninj1 blocking peptide to inhibit Ninj1, and this inhibited LPS‐induced pro‐inflammatory gene induction.^4^ Jennewein et al. reported Ninj1 blocking peptide administration attenuated sepsis in mice, and found Ninj1 blocking peptide reduced monocyte migration across endothelial cells. These previous studies reported the effects of Ninj1 blocking peptide on macrophages and endothelial cells with focus on inflammatory responses. We suggest that the effect of Ninj1 blocking peptide on TNF‐α/TNFR1‐induced cell death be comprehensively investigated to verify the therapeutic potential of Ninj1 blocking peptide targeting hepatocytes and that studies also be conducted to elucidate the molecular mechanism responsible for TNF‐α/TNFR1 complex II signal regulation by Ninj1 to determine whether Ninj1 is a proper therapeutic target and to develop therapeutic strategies that address liver diseases including ALF.

In conclusion, the results of our in vivo and in vitro studies demonstrate that lack of Ninj1 in hepatocytes ameliorates LPS/D‐gal‐induced ALF in mice by reducing TNF‐α/TNFR1‐induced cell death and suggest that inhibition of Ninj1 offers a potential means of treating ALF.

## AUTHOR CONTRIBUTIONS

**Min Woo Kim:** Data curation (lead); formal analysis (lead); investigation (lead); writing – original draft (lead). **Ju‐Hee Kang:** Data curation (equal); investigation (equal). **Hyun Jin Jung:** Data curation (supporting); investigation (equal). **Se Yong Park:** Data curation (equal); formal analysis (equal); investigation (equal). **Jong‐Ik Hwang:** Methodology (equal); resources (equal). **Je Kyung Seong:** Conceptualization (equal); methodology (equal); resources (equal). **Yeo Sung Yoon:** Conceptualization (equal); supervision (equal); writing – review and editing (equal). **Seung Hyun Oh:** Conceptualization (equal); funding acquisition (equal); supervision (lead); writing – review and editing (equal).

## CONFLICT OF INTEREST

The authors declare no conflict of interest.

## Supporting information


Appendix S1

Figure S1

Figure S2

## Data Availability

Datasets are available from the corresponding author upon reasonable request.
