# Supplementary material for: Deficiency of Ninjurin1 attenuates LPS/D‐galactosamine‐induced acute liver failure by reducing TNF‐α‐induced apoptosis in hepatocytes
Source: J Cell Mol Med. 2022 Sep 7;26(20):5122–34. doi: 10.1111/jcmm.17538 (PMC9575046; doi:10.1111/jcmm.17538)
Supplement: Supplementary file 1 — Appendix S1 [file JCMM-26-5122-s002.docx]

Primary antibodies

Primary antibodies for Western blotting were obtained from several different vendors, as follows: poly (ADP-ribose) polymerase (PARP) (Cell Signaling Technology, Danvers, MA, USA, #9542), caspase 3 (Cell Signaling Technology, #9662), caspase 9 (Cell Signaling Technology, #9508), human cleaved caspase 8 (Cell Signaling Technology, #9496), mouse cleaved caspase 8 (Cell Signaling Technology, #8592), pp65 (Cell Signaling Technology, #3033), phospho-extracellular-signal-regulated kinase (pERK) (Cell Signaling Technology, #4370), phospho-c-Jun N-terminal kinase (pJNK) (Cell Signaling Technology, #9251), pp38 (Cell Signaling Technology, #4511), TNFR1 (Cell Signaling Technology, #3736), c-IAP1 (Cell Signaling Technology, #4952), X-linked inhibitor of apoptosis protein (XIAP) (Cell Signaling Technology, #2042), glyceraldehyde 3-phosphate dehydrogenase (GAPDH) (Merck Millipore, Darmstadt, Germany, CB1001), caspase 8 (R&D Systems, Minneapolis, MN, USA, AF705), human Ninj1 (R&D Systems, AF5105), and mouse Ninj1 (AbClon, Seoul, Korea). All primary antibodies for western blotting were diluted 1:1,000, except for murine Ninj1 antibody (dilution 1:3,000). Primary antibody against cleaved caspase 3, which was used for immunohistochemistry (IHC), was obtained from Cell Signaling Technology.

Enzyme-Linked Immunosorbent Assay (ELISA)

Serum TNF-α levels were determined by ELISA using the DuoSet^®^ mouse TNF-α ELISA kit (R&D Systems, Minneapolis, MN, USA). Briefly, goat anti-mouse TNF-α capture antibody was coated on a plate overnight and blocked. Mouse TNF-α standards or serum samples were then incubated in each well for 2 h, and biotinylated detection antibody was added and incubated for 2 h. After incubation with streptavidin-horseradish peroxidase for 20 min, development was performed by adding substrate solution for 20 min. After terminating reactions using stop solution, optical densities were measured using a microplate reader (450 nm). Concentrations were calculated using the 4-parameter logistic method.

Primary cell isolation

Primary hepatocytes were isolated from 10- to 12-week-old male mice. Briefly, mice were anesthetized with isoflurane, and livers were exposed and perfused through the portal vein with EDTA buffer to remove all blood, followed by digestion buffer containing 0.02% DNase I and 37 mg of collagenase I per 50 ml of Hank's balanced salt solution (Welgene, Daegu, Korea) at 37℃. After digestion, liver was carefully collected, and chopped several times. Cell suspensions were filtered through a 70 μm cell strainer and centrifuged at 500 rpm, for 5 min at 4℃. To isolate viable hepatocytes, cell pellets containing hepatocytes were processed by gradient centrifugation at 1,600 rpm for 10 min at 4℃ using 40% Percoll reagent (Sigma-Aldrich, St. Louis, MO, USA). After one wash with PBS cell pellets were resuspended in low glucose DMEM supplemented with 10% FBS and penicillin/streptomycin (Welgene, Daegu, Korea) for further experiments.

MTT assay

To evaluate viability, cells were seeded on 96-well plates and incubated overnight. The next day, cells were treated with or without TNF-α/ActD for 18 h or 36 h. MTT solution (20 μl; 2.5 mg/ml) was then added to each well and incubated for 2 h. After removing medium, 100 μl of dimethyl sulfoxide was added to each well, and absorbances were measured using a microplate spectrophotometer at 562 nm.

Western blot analysis

Cells or tissue specimens were lysed using lysis buffer (20 mM Tris-HCl (pH 7.6), 1 mM EDTA, 140 mM NaCl, 1% NP-40, 1 mM sodium fluoride, and 1 mM sodium vanadate). Protein concentrations were determined using a bicinchoninic acid protein assay kit (Pierce, IL, USA). Protein samples were prepared by adding SDS-sample buffer and boiling for 5 min at 95℃, and SDS-polyacrylamide gel electrophoresis was then used to separate proteins by size. Proteins were transferred to polyvinylidene fluoride membranes (Pall, NY, USA), which were then blocked in 5% skim milk and washed with TBS-T (0.1% Tween 20). Primary antibodies were conjugated overnight at 4℃, and then secondary antibodies were added and incubated for 1 h at room temperature. Detection was performed using enhanced chemiluminescence substrate (AbClon, Seoul, Korea).
