# Supplementary figures and images for: Deficiency of Ninjurin1 attenuates LPS/D‐galactosamine‐induced acute liver failure by reducing TNF‐α‐induced apoptosis in hepatocytes
Source: J Cell Mol Med. 2022 Sep 7;26(20):5122–34. doi: 10.1111/jcmm.17538 (PMC9575046; doi:10.1111/jcmm.17538)

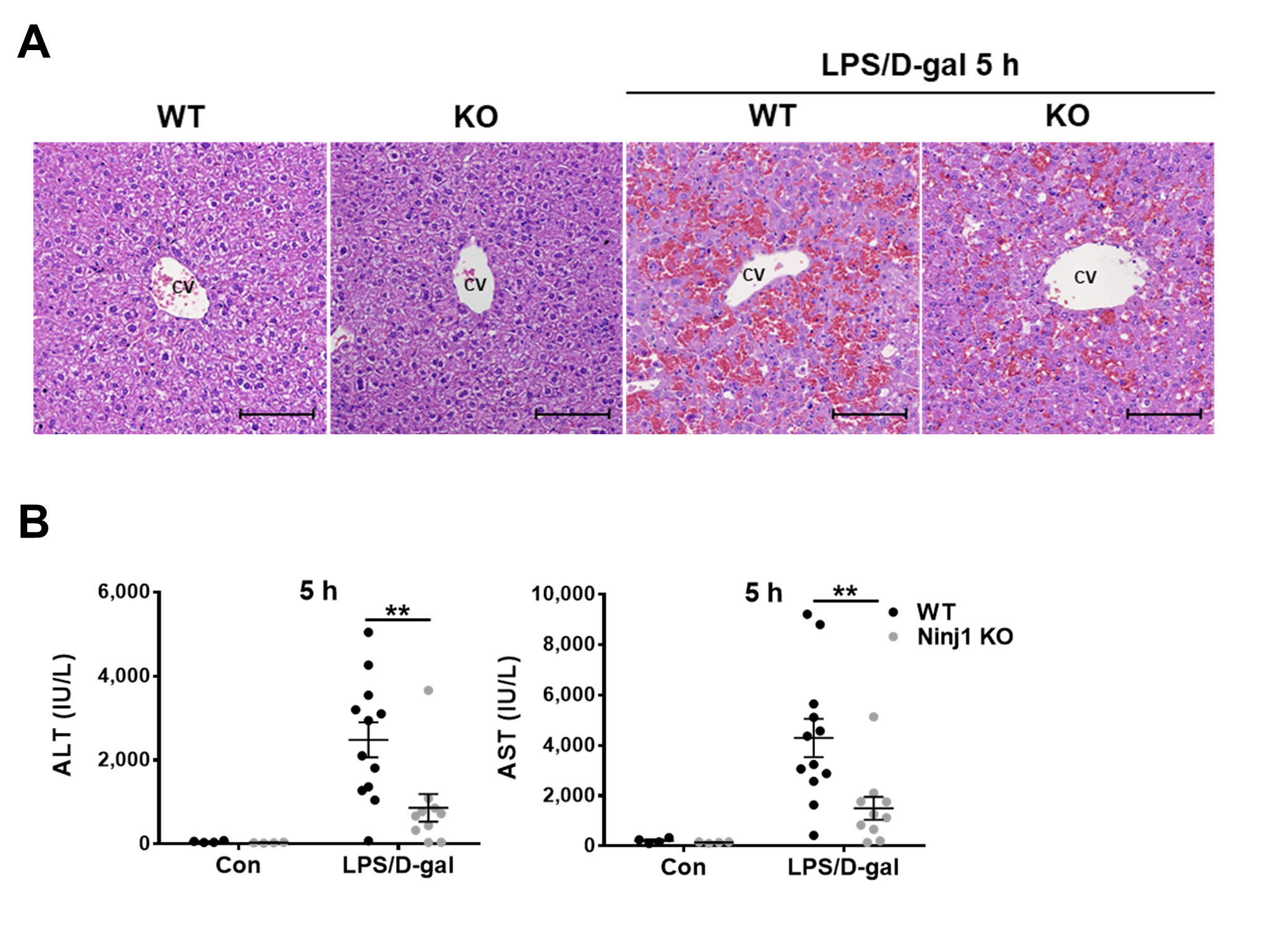

Supplement: Supplementary file 2 — Figure S1 [file JCMM-26-5122-s001.tif]

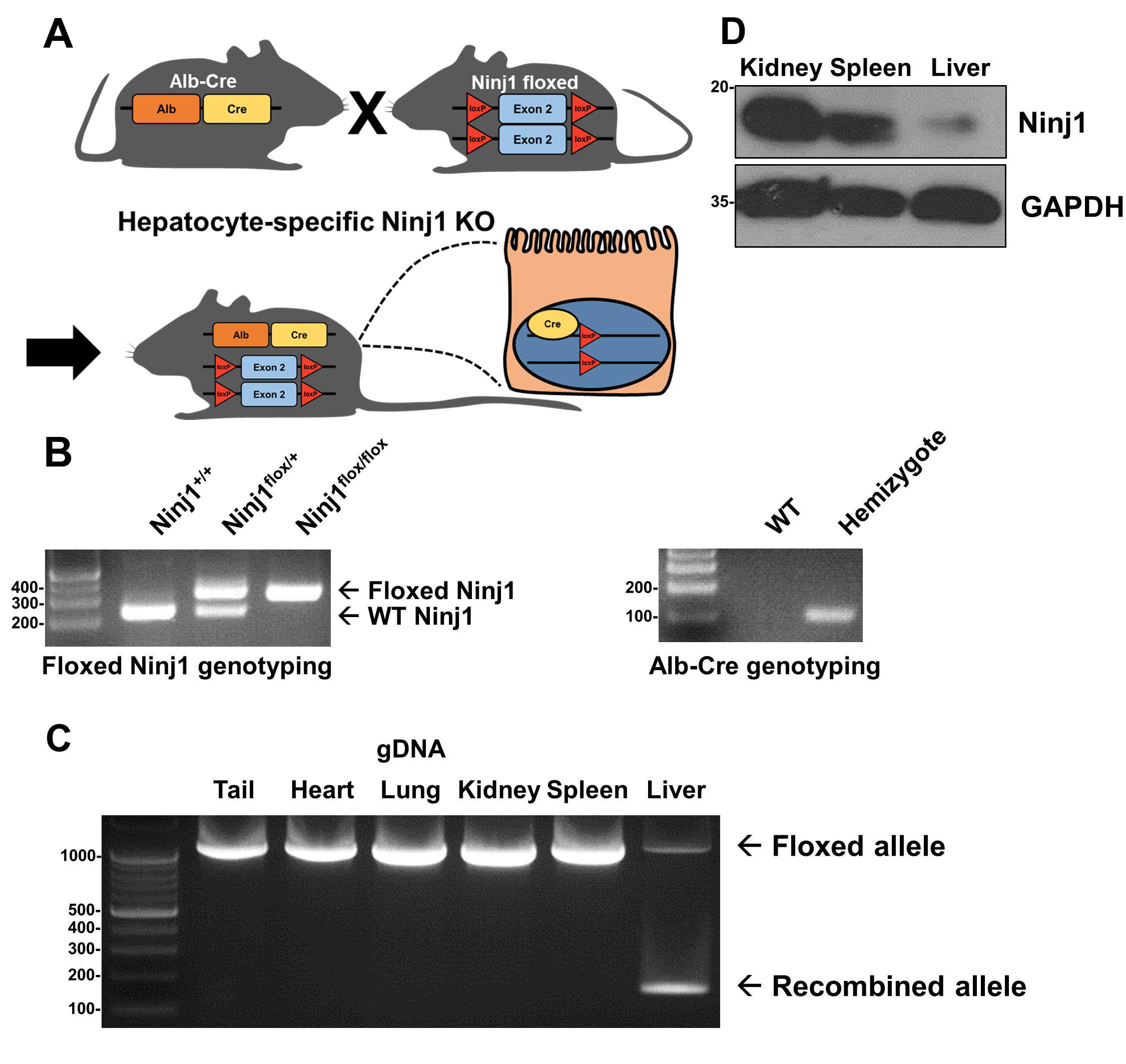

Supplement: Supplementary file 3 — Figure S2 [file JCMM-26-5122-s003.tif]
